# Innovation to Immune: Empirical Evidence From COVID-19 Focused Enterprise Surveys

**DOI:** 10.3389/fpsyg.2022.850842

**Published:** 2022-03-30

**Authors:** Karamat Khan, Sheng Liu, Baowei Xiong, Leihao Zhang, Chuntao Li

**Affiliations:** ^1^School of Economics, Henan University, Kaifeng, China; ^2^School of Business, Anhui University, Hefei, China; ^3^School of Economics, Zhongnan University of Economics and Law, Wuhan, China; ^4^School of Statistics and Mathematics, Zhongnan University of Economics and Law, Wuhan, China

**Keywords:** firm innovation, COVID-19, recession, survival, adaptability

## Abstract

The recent COVID-19 crisis caught many by surprise. Yet some firms were better prepared to weather the downturn than others. Using a comprehensive data set that observes over 15,000 firms in 27 countries, including several developing countries, shortly before and after the pandemic, we document that pre-crisis innovation affected firm’s survival odds and performance thereafter. The results show that innovative firms are less likely to close and perform better than non-innovators during the pandemic crisis. Innovative firms are also more optimistic about the future than non-innovators. Our results further indicate that firm’s adaptability mediates the relationship between innovation and survival outcomes. The study finding shows that innovative firms are more likely to introduce new products, remote work arrangements, increase delivery, pivoting, and online activities than non-innovators during the pandemic crisis.

## Introduction

Several infectious outbreaks have hit the world during the last two decades, including the Severe Acute Respiratory Syndrome (SARS) outbreak 2002–2004, the Swine Flu pandemic 2009–2010, and the Ebola virus epidemic 2013–2016. About 244 million people have been infected by the recent pandemic, COVID-19, with over 4.96 million fatalities are reported by the end of October 2021.^1^ The COVID-19 pandemic is regarded as a grave emergency for public health and an unprecedented blow to the economy (Chundakkadan et al., 2020; Fu and Shen, 2020; Khan et al., 2020; Narayan, 2020). The pandemic has caused a global economic crisis unlike any other in terms of cause, scale, severity, and policy response (Reinhart, 2020; Khan et al., 2021).

The enormity of the COVID-19 pandemic caught several businesses off guard. Many find themselves struggling for their survival. Fortune, on the other hand, favors the prepared mind. Some businesses are in a better position to cope with the recession than others. For instance, Grover and Karplus (2021) notes that firms with structured management practices are more resilient to the downside impacts of the COVID-19 crisis. Li et al. (2020) finds that firms with a strong corporate culture outperform their counterparts without strong culture amid the COVID-19 crisis. Guo et al. (2020) showed that digitalization has helped firms cope with the crisis. Ding et al. (2020) showed that firms with strong pre-crisis financial conditions and corporate social responsibility (CSR) activities faced a slight drop in stock prices during the COVID-19 induced crisis.

This paper contributes to the ongoing effort of exploring firm characteristics that foster resilience during the crisis. Previous research has extensively studied the link between innovativeness and firm survival (Cefis, 2005; Cefis and Marsili, 2006, 2019; Fontana and Nesta, 2009; Sidorkin and Srholec, 2014; Zhang et al., 2018; Ugur and Vivarelli, 2021). However, with a few exceptions, our understanding of this relationship is framed mainly in the absence of a major economic crisis. Given the important and multifaceted role of innovation, it is interesting to investigate whether or how innovation is linked with firm survival during the crisis caused by the COVID-19 pandemic. This article aims to fill this gap in the literature. To the best of our knowledge, this study is one of the first to demonstrate that innovative firms are more resilient to the economic downturn of the COVID-19 pandemic. The primary objective of the present investigation is to examine whether innovative firms are more likely to outperform non-innovators during the COVID-19 pandemic in terms of survival and other performance indicators. The study’s secondary goal is to investigate whether firm adaptability capability is a potential mechanism for innovators to affect their odds of survival.

We make several distinct contributions to the literature on the innovation–survival relationship. First and foremost, we deepen our understanding of innovation’s role in determining firm survival during the recent COVID-19 crisis. We contribute to the innovation literature, document a positive association between innovativeness and firm survival outcomes during the recent COVID-19 crisis. The results of our analysis are in line with earlier empirical studies on this topic (Yıldız et al., 2014; Fernandes and Paunov, 2015; Oura et al., 2016; Cefis et al., 2020; Fiorentino et al., 2020; Ugur and Vivarelli, 2021), and supports theoretical arguments asserting a positive relationship between innovation and firm survival (Schumpeter, 1943; Porter, 1980; Cohen and Klepper, 1996a,b; Teece et al., 1997; Zahra and George, 2002).

Furthermore, the ability to innovate is a strength and a capability that helps businesses become more suited and adaptable to their surroundings (Cefis et al., 2020). During extreme recessions, the innovation–survival relationship remains valid, with innovators having a better probability of surviving than non-innovators owing to their greater ability to adapt and adjust to the changes in the external environment (Cefis and Marsili, 2019; Cefis et al., 2020; Guo et al., 2020; Gupta, 2020; Li et al., 2020). We empirically tested this proposition and found that when firms were taken by surprise by the COVID-19 crisis, innovative firms quickly responded and adjusted to the new environment by introducing new work arrangements, products, enhancing online activity and readjusting their production, services, and delivery services. Third, to our knowledge, we consider the largest set of “survival outcomes,” such as the firm exit of market, performance, and future expectations amid crisis-like situations. Fourth, most of the accumulating evidence on firm characteristics and survival is based on single country settings or small sample sizes. However, our study uses a large, representative sample of 15,451 firms from 27 economies, enabling us to generalize our results.

Lastly, our study uses both product and process innovation in determining “survival outcomes.” Prior research on the innovation–survival mainly focused on the implications of product innovation and paid less attention to the impact of process innovation (Cefis and Marsili, 2012). This is possibly because process innovation is typically considered a “second-order innovative activity, a rather dull and unchallenging cousin of the more glamorous product innovation” (Reichstein and Salter, 2006). However, as with product innovation, process innovation is an important dynamic of competition in an industry. In bad times, firms could reduce marginal cost through process innovation and consequently lower selling prices to attract a large customer base (Gupta, 2020). Regressions are estimated using ordinary least squares (OLS) for continuous variables, with probit models used in the case of binary dependent variables.

The rest of the paper is structured as follows: section “Theoretical and Empirical Background” provides an overview of the empirical and theoretical literature on the innovation survival connection, followed by hypothesis development. Section “Data and Methodology” describes the study data set, variables, summary statistics, and methodology. In section “Results” results are presented while section “Discussion and Conclusion” summarizes and discusses the main findings and concludes the paper with policy implications.

## Theoretical and Empirical Background

The dominant view in the empirical literature supports a positive relationship between firm innovation–survival and performance thereafter. For instance, Rosenbusch et al. (2011) carry out a systematic review on the relationship between firm innovation and performance in small and medium-sized enterprises. Their study concludes that indeed innovation is positively and significantly linked with firm performance. The meta-analysis by Song et al. (2007) also examined a number of empirical studies exploring the relationship between innovation and firm performance. Their analysis found that over two-thirds of the empirical studies they review have a positive relationship between innovation and performance. However, both of these studies also reported that the link between innovation–performance might be context-dependent and relationship strength may decrease when input innovation measures are used in the analysis instead of output innovation. More recently, Ugur and Vivarelli (2021) builds on the existing reviews until 2020 and updates the evidence synthesis. Drawing on meta-analysis tools, they establish positive implications of innovation for firm survival and productivity.

Fiorentino et al. (2020) investigates the systematic differences in growth between innovative and non-innovative start-ups. Data from more than 34,000 firms located in Italy are analyzed by regression modeling and propensity score matching. The results show that differences in growth can be explained by the different levels of innovation. Moreover, the results show that input innovation is more beneficial than output innovation for firm growth. Yıldız et al. (2014) using questionnaire-based data analyze the impact of innovation on firm performance. The authors find that innovation is significantly positively related to firm performance. Oura et al. (2016) found a positive contribution of innovation capacity on SME’s export performance located in emerging country.

Turning more specifically to the innovation–survival relationship, Fernandes and Paunov (2015) used a data set on Chilean manufacturing plants and their products during 1996–2006. The study used a hazard or duration model where the dependent variable is the survival spell between plant entry and exit. The study findings support the innovation–survival link. Moreover, they note that risk plays a significant role in determining the innovation–survival relationship. For instance, the odds of survival were higher for plants with diversified sources of revenue, multi-product innovations, and lower market risks. In a series of research papers, (Cefis, 2005; Cefis and Marsili, 2006, 2011, 2012) investigated the impact of innovation on firm survival in a sample of Dutch manufacturing firms. Using community innovation surveys, they found strong evidence that innovation is significantly and positively related to firm survival prospects. More recent work by Zhang et al. (2018) explored the relationship in a sample of Chinese high-tech firms. As gauged by patents and innovation efficiency, authors conclude that innovation improves survival odds in Chinese high-tech firms. Colombelli et al. (2013) examined how aspects of a firm’s patent stock influence survival. The study uses a sample of 85,070 French manufacturing firms covering a time span ranging from 2001 to 2011. Findings revealed that innovation increases firm survival prospects. Further support is given by Esteve-Pérez and Mañez-Castillejo (2008) study that use a sample of manufacturing firms to test hypothesis derived from the resource-based theory of the firm. These researchers assert that the development of firm capabilities *via* R&D enhances survival chances. Wagner and Cockburn (2010) use a representative sample of Internet-related firms that made an initial public offering on the NASDAQ to examine the impact of patenting on survival prospects. They find that patenting is positively related to firm survival. Coad and Guenther (2013) use data for German machine tool manufacturers in the post-war era to examine the relationship between age and diversification patterns measured by introducing new products in familiar and new markets. Among other things, they note that innovation reduces the risk of firm exit. Dai et al. (2020a) investigate the impacts of innovation on firm survival in the export market. The study employs panel data from 2000 to 2010 of Chinese firms to explore the relationship. These authors report a statistically significant and positive impact of innovation on firm export survival. Helmers and Rogers (2010) examines the impact of innovation on survival of new firms. The evidence suggests that innovation is negatively related to failure rate of sampled firms.

There are also a number of studies investigating innovation–survival links during the severe economic crisis. More recent work by Adam and Alarifi (2021) examine the relationship between SME’s innovation practices and survival outcomes during the COVID-19 pandemic. Using a data set from a survey with 259 Saudi Arabian SME’s, the authors note that innovation is significantly positively related to firm performance and likelihood of business survival. Cefis et al. (2020), using a sample of 6,542 Italian manufacturing firms, explored the conditional impact of financial constraints on the firm innovation–survival relationship during the economic downturn. They reported that innovative firms have a higher likelihood of surviving the crisis than non-innovators. Moreover, a firm’s financial constraints do not eliminate the survival premium, even though they mitigate it. Gupta (2020) investigated whether innovation helped Spanish manufacturing firm to shield from the adverse shocks of 2008 financial crisis. His findings showed that innovative firms were more resilient to the downside impacts of the crisis compared to non-innovators. The author elucidated that innovative firms have a higher capability to adjust to rapid changes in the external environment. Li et al. (2020), while using data for 2,894 U.S. firms, examined the relationship between firm overall exposure to COVID-19 crisis, stock market performance, and corporate culture. The study used a comprehensive measure of corporate culture covering innovation. Their findings revealed that firms with strong corporate culture performed better than those with weak culture during the COVID-19 crisis. Ding et al. (2020) used a sample of 6,000 firms across 56 economies to examine the relationship between firm pre-crisis characteristics and stock price reactions to COVID-19 cases. Among other things, it was found that firms with more CSR activities (including green innovation) were resilient to the pandemic induce drop in stock prices.

Nevertheless, Sidorkin and Srholec (2014), based on the data sets obtained from the World Bank Enterprise Surveys and Financial Crisis surveys, examined the impact of innovation on their odds of survival and performance thereafter. Their study finds that firms which excessively innovate before the crisis are more likely to exit the market than cautious innovators. However, their analysis is primarily based on innovation intensity rather than those who innovate and do not innovate.

Economists explain the closure of firms during recessions with Schumpeter’s (1943) creative destruction theory, where during recessions, less innovative firms are the ones to exit the market. According to Schumpeter (1943), innovation “strikes not at the margins of the profits and the outputs of the existing firms but at their foundations and their very lives.” More recently, this view was endorsed by Baumol (2002) and called innovation activity “a life and death matter for the firm.”

**Figure 1:**
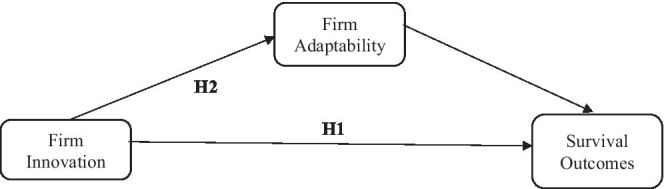
The hypothesized model.

Drawing insights from creative destruction theory, a range of theoretical arguments also point to a positive relationship between innovation and subsequent survival. In addition to making entry possible, innovation increases firm’s market power, improve their ability to escape competition, reduce their production costs, improve dynamic capabilities, and lead to enhanced absorptive capacity (Schumpeter, 1943; Porter, 1980; Cohen and Klepper, 1996a,b; Teece et al., 1997; Zahra and George, 2002). There is evidence that having introduced an innovation enhances the probability of firm survival persistently over time, years after the innovation has taken place (Cefis and Marsili, 2006). Innovation is a valuable and appropriable resource that generates a sustained positional advantage for the firm in a competitive context (Barney, 1991). Innovation is also a capability because firms learn how to recognize and exploit commercially novel opportunities and how to solve problems, as they engage in the process of introducing novel products, processes, or practices (Nelson and Winter, 1982). This cumulatively built knowledge, which includes skills, competences, and practices is stored in routines (Nelson and Winter, 1982) and generates persistence in innovative capabilities and outcomes (Cefis, 2003). Such a learning process enhances organization flexibility and adaptability to future changes, either internal or external to the firm. Hence, innovation as a resource and a capability contributes to create both a “positional advantage,” because innovative firms are rewarded through market selection in view of asymmetries in some dimension of performance (or fitness), and an “adaptive advantage,” because firms with superior innovative capabilities can change their relative position in the distribution of performance, through learning and the exploration of new opportunities. While the overall importance of innovation for survival is well established in the literature, little is known about the distinctive contribution of positional and adaptive mechanisms of survival. Given these theoretical and empirical antecedents, we propose following hypothesis (Figure 1):

*H1*: Firm innovation has a significant positive impact on firm survival outcomes during the COVID-19 pandemic crisis.

*H2*: Firm adaptability capability mediates the relationship between innovation and survival outcomes.

## Data and Methodology

### Data

We use a unique and novel firm-level data set derived from two sources. The first data set is obtained from the World Bank Enterprise Survey (WBES), covering 1,69,938 firms in 146 countries. The WBES data set uses standardized monitoring instruments to assess the business environment of each economy and its effect on business operation and performance. The data set includes firms from both services and manufacturing sectors and conducted before the COVID-19 pandemic from 2006 to 2020. Therefore, our pre-crisis firm-level innovation variables and control variables come from the WBES data base. The second data set is the World Bank COVID-19 enterprise follow-up surveys (CEFS). The CEFS is also a survey-based data collected from firms included under the sample frame of the recently conducted WBES for the same countries. The CEFS investigates how the COVID-19 pandemic has affected global businesses, particularly SMEs, in developed and developing economies. The CEFS looks into the impact of COVID-19 on businesses and the subsequent changes they make as a result, such as sales, growth, jobs, financial access, government assistance, and expectations resulting from the pandemic. We use the CEFS database to construct our dependent variables of the study. For data on stringency lockdown during the COVID-19 pandemic, we rely on Our World in Data.^2^

After obtaining the most updated and recently available data from sources, we combine the WBES data set with the observation of the same firm in CEFS by their unique identification “idstd.” WBES cover a large number of countries, but the follow-up COVID-19 surveys are conducted only in 36 of them. Thus, the initial sample size and country coverage are dictated by the CEFS. For some countries covered in the CEFS, follow-up surveys are conducted in two waves, of which the first took place in June/July 2020 and the second in September/December 2020. Our analysis includes only the latest available COVID-19 enterprise surveys for these countries, reducing our initial sample size from 27,686 to 19,209 firms in 36 countries. We further decided to limit the sample to the WBES surveys concluded in 2019 and onwards. Therefore, our final sample gives a glimpse of the firm condition just before and after the onset of the pandemic (approximately 6 months to 1.5 years apart). The final sample of the study consists of 15,451 firms in 27 countries.

### Variables

This subsection discusses variables used in the empirical analysis. This study is intended to investigate the impact of pre-crisis innovation on firm survival outcomes during the COVID-19 induced crisis. Apart from dependent and independent variables, we employ a range of control variables for the rigors test of the innovation–survival/performance hypothesis. Firm survival indicators (closure/performance/expectations) are our dependent variables. We use the World Bank COVID-19 enterprise follow-up surveys to construct these variables.

#### Dependent Variable: Firm Survival

This study constructs firm survival variables based on three categories that are firm closure, performance, and future expectations. Our first survival indicator is closure variable is “Close1” a dummy variable that takes the value of one if “firms are confirmed to have permanently closed since the COVID-19 outbreak” otherwise zero. The second closure indicator is “Close17” a dummy variable that takes the value of one if “firms are confirmed or assumed permanently closed since the COVID-19 outbreak” otherwise zero. “This includes both the firms that selected permanently closed in question ‘Close1’ and the firms that could not be contacted directly, but for which the information collected during the fieldwork suggests that they are likely to have closed permanently.” Third, the closure variable is “Close19” measured by “firms that are confirmed to have permanently closed since the COVID-19 was declared by the WHO as a pandemic, counting all the closures since March of 2020” otherwise zero. Firm performance is measured by “CFshort” which is a binary variable taking a score of one if “firms experiencing decreased liquidity or cash flow availability since the COVID-19 outbreak” otherwise zero; “Sales” is an alternate performance indicator constructed by “the average percentage change in monthly sales compared to 1 year ago.” The firm expectation is proxied by “WeeksSur” which is the logarithm of 1 plus the firm’s answer to “average duration of establishment survival in weeks if sales were to stop and assuming the current cost structure.” We also proxied firm expectation by a dummy variable “NeverReco” which takes the value of one if “firms that do not expect to ever return to the normal level of the workforce” otherwise zero.

#### Independent Variable: Firm Innovation

In the empirical literature, researchers used several different proxies to represent firm-level innovation. For instance, Balasubramanian and Lee (2008), Kotha et al. (2011), and Ren et al. (2015) represented innovation by “number of patent applications,” Hansen (1992), Sun and Du (2010), Chandran Govindaraju et al. (2013), and Love et al. (2014) used number of innovations and sales from new products, Withers et al. (2011) used innovation capability “degree to which a firm possesses resources and capabilities presumed necessary for innovation,” Bello-Pintado and Bianchi (2018) and Garriga et al. (2013), used “significance,” or radical and incremental innovation as a proxy for innovation, and yet a large number of researchers like Bratti and Felice (2012), Monreal-Pérez et al. (2012), Børing et al. (2016), Seenaiah and Rath (2018) and Spithoven et al. (2013) used a binary variable to gauge innovation “introduction of a new product/service/process or improved product/ service process.”

In this paper, data structure and enterprise scale are more suitable for measuring innovation with “Introduced New/Significantly Improved Product and process” because patent applications require long-term technology accumulation and are suitable for the measurement of large and medium-sized enterprises. These measures are constructed from pre-crisis enterprise surveys. The question of innovation in the enterprise survey asks managers or owners of the firms “During the Last Three Years, Establishment Introduced New/Significantly Improved Product.” An affirmative answer to this question takes the value of one, otherwise zero. Similarly, the enterprise survey also asks whether “During the Last Three Years, Establishment Introduced New/Significantly Improved Process.” An affirmative answer to this question takes the value of one, otherwise zero.

#### Mediating Variable: Adaptability

We also investigate the mechanisms through which innovation practices may affect survival outcomes. The study is based on the premise that innovative firms are quickly adjusting/adapting to new changes in the external environment, affecting firm survival indicators. We focus on five categories representing major adaptation in response to the crisis. We use the CEFS data set to construct these variables. For instance, “Pivoting” is a dummy variable that takes the value of one if “firms adjust or convert their production or services” otherwise zero. Similarly, “Covidps” takes the value of one if “firms introduced new product or service in response to COVID-19”; “Online” takes the value of one if “firms started or increased online business activity”; “Delivery” takes the value of one if “firms started or increased delivery of goods, services or carryout”; “Remote” takes the value of one if “firms started or increased remote work” otherwise zero for all these statements.

#### Control Variables

We control for a set of firm characteristics, namely, firm size (small < 20, medium 20–99 employees and large > 100), training (a dummy variable that takes the value of one if enterprise provided “formal training programs for permanent, full-time employees in last fiscal year”) as a measure for human capital quality. We use the (log of) labor productivity, measured by the ratio of real sales to the total workforce, as a proxy for productivity, expressed in 2005 USD. Firms facing an obstacle to access finance (Finobs) are more likely to exit the market during the crisis. We control for access to finance by the degree of financial obstacle during the pre-crisis period. Quality “captures the capability to conform to international standards of production and, thus, represents the production facet of technological capability” (Sidorkin and Srholec, 2014). The variable takes the value of one if “firm had an internationally recognized quality certification, for example, ISO 9,000, 9,002 or 14,000” otherwise zero. Age is measured by the natural logarithm of firm age. Managerial experience is proxied by the natural logarithm of the “top manager number of years of experience working in this sector.” Website is a dummy variable represented by the value of 1 if “establishment has its own website” Website is generally used to communicate with customers and suppliers, which captures external communication capability mediated by the INTERNET. Therefore, feeds the idea of Lundvall (1988) that reducing the information asymmetry between customers and producers is vital to innovation. Foreign ownership represents a dummy variable that takes the value of one if “equal to more than 10 percent of the firm owned by private foreign individuals, companies, or organizations.” An exporter is also a dummy variable that takes the value of one if the firm exports, zero otherwise. Female is a dummy variable which takes the value of one if the top manager is female, otherwise zero. The capital city is a dummy variable with the value of one if the firm is located in the capital city, zero otherwise; hence, it controls for advantages to firms operating in the urbanized economies. Stringency lockdown is a “government response stringency index” that measures the stringency of government responses (such as school closures, workplace closures, and travel bans) to COVID-19. The value of the index lies between 0 and 100, where higher means more stringent measures. We take an average stringency score for the months in which the COVID-19 surveys are conducted.

Additionally, our key results are likely to be influenced by many regional policies introduced within a country for the pandemic.^3^ We use regional dummy variables in our model to include its effect. Finally, we also control for unobserved industry-level heterogeneity by including industry dummies. In all the estimations, the standard errors are clustered at the regional level to account for the bias from the correlation between all observations within each region.^4^

### Empirical Approach

The next step is to empirically examine how innovation practices, as measured before the onset of COVID-19, is linked to a firm’s post-COVID-19 survival, expectations, and performance. We use regression analysis to investigate the relationship between innovation and a vector of seven survival outcomes, as defined in the previous section. To our knowledge, this is the most comprehensive set of COVID-19 outcomes ever considered in a study. Moreover, we also examine the mechanisms through which innovation may influence firm outcomes. The firm’s adaptability to the crisis proxied by adjusting product and services, delivery, remote working, online activity might be potential mechanisms. We use the ordinary least squares method for the continuous dependent variables, while the probit regression approach is employed for binary dependent variables. Control variables are the same in all regression specifications.

We understand that our regression results could be affected by the endogeneity issue. Reverse causality, simultaneity, and omitted variables are potential sources of endogeneity. We believe that reverse causality and simultaneity are unlikely to influence the regression outcomes in our sample for two reasons. First, the COVID-19 pandemic was an entirely unexpected event, which was not possible to predict prior to the outbreak in Wuhan in early 2020. The dependent variables in our study are the outcomes of this exogenous event. Second, the dependent variables are taken from the COVID-19 follow-up surveys conducted after the onset of the pandemic, while the innovation and other control variables are taken from the pre-crisis WBES. We try our best to minimize the effect of omitted variables in our data. Our regressions involve 13 firm-level control variables and region-industry fixed effects in addition to our core variable innovation. As a result, we expect the omitted-variable problem to be less of a concern in our case.

## Results

### Descriptive Statistics

Table 1 provides a brief overview and description of the variables used in our regression analysis. It shows that from 2 to 14.1% of firms permanently closed during the pandemic, based on three closure measures. The table also reports that 66.4% of firms experienced a decrease in liquidity since the COVID-19 pandemic started and a decline of 23.12% in monthly sales on average. The large differences in sales ranging from −100 to 300% indicate that some firms benefitted from the crisis. Moreover, firms were asked about their perception of future prospects. The statistics show that 1.5% of firms were uncertain about future recovery in terms of workforce. If sales stopped, firms might survive for another 5.62 weeks on average.

**Table 1:** Descriptive statistics.

Variables	Obs.	Mean	SD	Min	Max
*Dependent Variables*					
Close1	15,449	0.045	0.207	0	1
Close17	15,449	0.141	0.349	0	1
Close19	15,449	0.020	0.141	0	1
CFshort	11,140	0.664	0.472	0	1
Sales	10,708	−23.125	30.168	−100	300
NeverReco	10,038	0.015	0.123	0	1
WeeksSur	9,084	1.890	0.870	0	6.256
*Independent Variables*					
Product	15,322	0.262	0.439	0	1
Process	15,299	0.154	0.361	0	1
*Channels: Adaptability Variables*					
Pivoting	11,423	0.386	0.487	0	1
Online	11,079	0.269	0.443	0	1
Delivery	11,440	0.238	0.426	0	1
Remote	11,366	0.330	0.470	0	1
Covidps	4,445	0.180	0.384	0	1
*Controls Variables*					
*Firm size*					
Small	15,451	0.443	0.497	0	1
Medium	15,451	0.335	0.472	0	1
Large	15,451	0.222	0.416	0	1
Training	15,312	0.347	0.476	0	1
Labor Productivity	13,847	10.030	1.632	0.873	17.661
Obstacle to Access Fin.	15,111	1.192	1.219	0	4
Quality	15,113	0.279	0.449	0	1
Firm Age	15,257	2.871	0.678	0	5.313
Manager Experience	14,950	2.837	0.727	0	4.248
Website	15,415	0.662	0.473	0	1
Foreign	15,175	0.102	0.303	0	1
Exporter	15,187	0.233	0.423	0	1
Female Manager	15,417	0.184	0.388	0	1
Capital City	14,850	0.167	0.373	0	1
Stringency Index	15,102	61.645	15.649	16.670	80.725

The below table shows that a considerable number of firms in our sample reported product innovation (about 26.2%) and process innovation (about 15.4%) prior to the crisis period. Yet, a large number of firms adopted measures in response to the crisis. For instance, 38.6% of firms adjusted their product and services in response to the crisis; 26.9% of firms started or increased online activity; remote work arrangements (about 33% of firms); delivery of goods/services (about 23.8% of firms), introduced products/services (about 18%). In terms of firm characteristics, 44.3% of the firms are small, remaining are medium-sized firms (33.5%) and large-sized firms (22.2%). The bottom part of the table also provides descriptive statistics of other firm characteristics that we controlled in this empirical analysis along with the stringency of lockdown, which is a country-level indicator.

### Firm Innovation–Survival Relationship During COVID-19 Pandemic Crisis

We first investigate whether the innovation–survival link holds during the COVID-19 crisis. Based on various categories of survival variables, this question is further divided into three parts. Are innovative firms less likely to close than non-innovators during the COVID-19 crisis? Are innovative firms performing better than non-innovators during the COVID-19 crisis? Are innovative firms more pessimistic about the future than non-innovators during the COVID-19 crisis? The study uses pre-crisis firm-level innovation (product/process) as the main independent variable to address these questions. Further, we are also curious about the channels through which innovation may affect survival. Therefore, we examined whether innovative firms are more likely to adapt than non-innovators in response to the COVID-19 crisis. We selected five variables: pivoting, increased online activity, remote work arrangements, delivery, and introduction of new products as the firm’s adaptation response to the COVID-19 crisis. The empirical analysis uses the ordinary least square (OLS) method for the continuous dependent variables and the probit regression approach for the binary dependent variables.

The analysis begins by estimating a baseline regression where we regress survival variables with only pre-crisis firm-level product innovation (as in columns 1 to 7 of Table 2). Next, we re-estimate our results by including a range of firm-specific control variables and stringency of lockdown (as in columns 8 to 14 of Table 2). All regressions include industry and region fixed effects (coefficient estimates are not reported). We make several interesting observations based on estimated results presented in Table 2. First, we find that innovative firms are less likely to close permanently than non-innovation during the crisis. The relationship between pre-crisis product innovation and firm closure variables are negative and statistically significant across all estimated models except for “Close19” in column 10 which is statistically insignificant but carried a correct sign. From a performance perspective, we note that innovative firms have a lower likelihood of experiencing cash flow shortages and perform better than non-innovators in terms of sales during the COVID-19 induced crisis. Once again, these results are statistically significant and carried correct signs when we use baseline regression and all control variables. Further, we find that innovative firms may survive for a longer time when sales stop; and more optimistic about returning to normal level than non-innovators. However, the statistical significance weakens when we employ firm-level control variables and stringency of lockdown in the regression analysis. Overall, the coefficients carry a correct sign and provide support to our innovation–survival relationship hypothesis.

**Table 2:** Product innovation, closure, performance, and expectations.

	(1)	(2)	(3)	(4)	(5)	(6)	(7)	(8)	(9)	(10)	(11)	(12)	(13)	(14)
	Close1	Close17	Close19	CFshort	Sales	WeeksSur	NeverReco	Close1	Close17	Close19	CFshort	Sales	WeeksSur	NeverReco
Product	−0.401^***^	−0.227^***^	−0.337^***^	−0.083^***^	2.135^***^	0.058^***^	−0.188^***^	−0.218^***^	−0.157^***^	−0.153	−0.071^**^	1.747^**^	0.047^*^	−0.160^*^
	(0.0677)	(0.0360)	(0.0995)	(0.0314)	(0.7326)	(0.0198)	(0.0725)	(0.0741)	(0.0395)	(0.1060)	(0.0332)	(0.7501)	(0.0252)	(0.0873)
Medium								−0.150^**^	−0.011	−0.146^*^	−0.076^*^	1.730^**^	0.015	0.031
								(0.0592)	(0.0354)	(0.0866)	(0.0449)	(0.7024)	(0.0254)	(0.1030)
Large								−0.286^***^	0.107^**^	−0.514^***^	−0.203^***^	4.234^***^	0.065^**^	0.170
								(0.0839)	(0.0518)	(0.1602)	(0.0571)	(1.1366)	(0.0307)	(0.1250)
Training								−0.141^**^	−0.114^***^	−0.070	−0.029	−0.050	0.053^**^	−0.347^***^
								(0.0708)	(0.0397)	(0.0717)	(0.0326)	(0.6007)	(0.0215)	(0.1106)
Productivity								−0.073^***^	−0.026^*^	−0.076^***^	−0.105^***^	2.346^***^	0.051^***^	−0.106^***^
								(0.0173)	(0.0135)	(0.0183)	(0.0198)	(0.3099)	(0.0128)	(0.0336)
Access Fin.								0.070^***^	0.037^***^	0.027	0.034^**^	−0.659^**^	−0.022^**^	0.120^***^
								(0.0257)	(0.0126)	(0.0295)	(0.0163)	(0.2817)	(0.0098)	(0.0282)
Quality								0.081	0.082^*^	−0.065	−0.065	2.439^***^	0.005	0.003
								(0.0649)	(0.0457)	(0.1189)	(0.0431)	(0.9096)	(0.0233)	(0.0991)
Age								−0.106^**^	−0.104^***^	−0.083	−0.025	1.047^*^	0.034^*^	−0.017
								(0.0471)	(0.0296)	(0.0531)	(0.0268)	(0.5348)	(0.0195)	(0.0877)
Experience								0.027	−0.023	0.101	0.007	−1.128^**^	0.008	0.012
								(0.0489)	(0.0277)	(0.0756)	(0.0288)	(0.5607)	(0.0196)	(0.0845)
Website								−0.224^***^	−0.157^***^	−0.110	0.005	−0.203	0.106^***^	−0.000
								(0.0565)	(0.0396)	(0.0707)	(0.0437)	(1.0035)	(0.0310)	(0.0876)
Foreign								0.159^*^	0.159^***^	0.344^***^	−0.035	0.539	0.056^*^	0.032
								(0.0820)	(0.0538)	(0.1319)	(0.0481)	(0.9181)	(0.0334)	(0.1456)
Exporter								−0.065	−0.056	−0.079	−0.048	−0.932	0.111^***^	−0.150
								(0.0640)	(0.0393)	(0.1248)	(0.0469)	(0.9933)	(0.0278)	(0.1236)
Female Mang.								0.003	−0.013	0.207^***^	−0.025	−0.274	−0.132^***^	−0.016
								(0.0620)	(0.0373)	(0.0736)	(0.0379)	(0.8649)	(0.0276)	(0.1192)
Capital City								0.077	0.039	0.185^*^	0.216^***^	−1.841	−0.016	0.227^**^
								(0.1211)	(0.0791)	(0.1113)	(0.0564)	(1.3602)	(0.0474)	(0.1137)
Stringency Index								0.028^***^	0.018^***^	0.014^**^	0.002	−0.067	0.009^*^	0.011
								(0.0046)	(0.0031)	(0.0067)	(0.0045)	(0.0481)	(0.0049)	(0.0113)
Industry F.E.	Yes	Yes	Yes	Yes	Yes	Yes	Yes	Yes	Yes	Yes	Yes	Yes	Yes	Yes
Region F.E.	Yes	Yes	Yes	Yes	Yes	Yes	Yes	Yes	Yes	Yes	Yes	Yes	Yes	Yes
_cons	−0.647^***^	−1.662^***^	−1.189^***^	1.209^***^	−32.289^***^	1.846^***^	−2.856^***^	−2.081^***^	−2.538^***^	−2.143^***^	2.430^***^	−56.343^***^	0.277	−2.694^**^
	(0.1260)	(0.0619)	(0.2015)	(0.0734)	(1.3444)	(0.0407)	(0.2933)	(0.3178)	(0.3717)	(0.5072)	(0.3644)	(5.3833)	(0.3524)	(1.1675)
Observations	13,584	15,176	10,696	11,047	10,616	9,014	6,460	10,576	12,018	7,722	8,622	8,504	7,615	4,957
*R*^2^/Pseudo *R*^2^	0.0829	0.0783	0.1113	0.0999	0.2021	0.1111	0.0569	0.1012	0.0961	0.1139	0.0972	0.2045	0.1289	0.0904

*** Significant at 1%;

** significant at 5%;

* significant at 10%.

*Clustered standard errors in parentheses, errors are clustered at the regions level*.

We check whether our results are robust using an alternative proxy of firm-level innovation. We explore in this section the robustness of the findings using pre-crisis process innovation as an alternate measure of firm-level innovation. We estimate regressions where we include only process innovation as an explanatory variable and control for the region and industry fixed effects and then add all firm-specific control variables, including stringency of lockdown and region-industry fixed effects. As shown in Table 3, we find that innovativeness is associated with decreased firm closure, increased firm performance, and less pessimism. These results are statistically significant and signs are correctly specified (except in columns 10 and 13 of Table 3, where these results are insignificant but carry the correct sign). The results presented in Table 3 confirm our previous findings that innovative firms are more resilient to the economic downturn caused by the COVID-19 pandemic.

**Table 3:** Process innovation, closure, performance, and expectations.

	(1)	(2)	(3)	(4)	(5)	(6)	(7)	(8)	(9)	(10)	(11)	(12)	(13)	(14)
	Close1	Close17	Close19	CFshort	Sales	WeeksSur	NeverReco	Close1	Close17	Close19	CFshort	Sales	WeeksSur	NeverReco
Process	−0.343^***^	−0.179^***^	−0.204^**^	−0.138^***^	2.880^***^	0.068^**^	−0.345^***^	−0.175^**^	−0.130^**^	−0.083	−0.086^**^	1.915^**^	0.031	−0.397^***^
	(0.0808)	(0.0478)	(0.0944)	(0.0378)	(0.9010)	(0.0265)	(0.1028)	(0.0800)	(0.0514)	(0.1075)	(0.0413)	(0.7644)	(0.0283)	(0.1363)
Medium								−0.151^***^	−0.014	−0.147^*^	−0.077^*^	1.758^**^	0.014	0.042
								(0.0583)	(0.0357)	(0.0865)	(0.0447)	(0.7000)	(0.0252)	(0.1029)
Large								−0.280^***^	0.105^**^	−0.512^***^	−0.206^***^	4.226^***^	0.065^**^	0.173
								(0.0837)	(0.0523)	(0.1590)	(0.0572)	(1.1363)	(0.0308)	(0.1248)
Training								−0.144^**^	−0.111^***^	−0.074	−0.027	−0.082	0.056^**^	−0.321^***^
								(0.0693)	(0.0390)	(0.0735)	(0.0319)	(0.5678)	(0.0216)	(0.1090)
Productivity								−0.074^***^	−0.026^*^	−0.076^***^	−0.104^***^	2.332^***^	0.051^***^	−0.109^***^
								(0.0171)	(0.0135)	(0.0180)	(0.0197)	(0.3123)	(0.0129)	(0.0340)
Access Fin.								0.069^***^	0.037^***^	0.026	0.033^**^	−0.645^**^	−0.022^**^	0.118^***^
								(0.0255)	(0.0128)	(0.0292)	(0.0161)	(0.2823)	(0.0099)	(0.0285)
Quality								0.088	0.082^*^	−0.065	−0.060	2.332^**^	0.006	0.025
								(0.0640)	(0.0461)	(0.1182)	(0.0430)	(0.9187)	(0.0227)	(0.1001)
Age								−0.108^**^	−0.105^***^	−0.079	−0.028	1.086^**^	0.033^*^	−0.016
								(0.0474)	(0.0297)	(0.0539)	(0.0269)	(0.5350)	(0.0193)	(0.0877)
Experience								0.025	−0.023	0.099	0.010	−1.233^**^	0.007	0.016
								(0.0488)	(0.0279)	(0.0761)	(0.0284)	(0.5522)	(0.0197)	(0.0857)
Website								−0.242^***^	−0.165^***^	−0.120^*^	0.002	−0.095	0.110^***^	−0.005
								(0.0568)	(0.0390)	(0.0711)	(0.0435)	(0.9919)	(0.0301)	(0.0855)
Foreign								0.155^*^	0.157^***^	0.344^***^	−0.031	0.472	0.058^*^	0.052
								(0.0813)	(0.0543)	(0.1295)	(0.0470)	(0.9230)	(0.0336)	(0.1467)
Exporter								−0.076	−0.053	−0.085	−0.047	−0.820	0.114^***^	−0.155
								(0.0668)	(0.0395)	(0.1235)	(0.0463)	(0.9925)	(0.0281)	(0.1230)
Female Mang.								0.001	−0.018	0.203^***^	−0.026	−0.252	−0.129^***^	−0.020
								(0.0614)	(0.0373)	(0.0729)	(0.0380)	(0.8637)	(0.0278)	(0.1207)
Capital City								0.075	0.037	0.186^*^	0.222^***^	−1.868	−0.014	0.236^**^
								(0.1225)	(0.0787)	(0.1110)	(0.0559)	(1.3586)	(0.0472)	(0.1201)
Stringency Index								0.028^***^	0.018^***^	0.015^**^	0.002	−0.062	0.009^*^	0.011
								(0.0053)	(0.0034)	(0.0069)	(0.0045)	(0.0511)	(0.0049)	(0.0115)
Industry F.E.	Yes	Yes	Yes	Yes	Yes	Yes	Yes	Yes	Yes	Yes	Yes	Yes	Yes	Yes
Region F.E.	Yes	Yes	Yes	Yes	Yes	Yes	Yes	Yes	Yes	Yes	Yes	Yes	Yes	Yes
_cons	−0.626^***^	−1.700^***^	−1.179^***^	1.204^***^	−32.005^***^	1.856^***^	−2.857^***^	−2.044^***^	−2.540^***^	−2.187^***^	2.401^***^	−56.197^***^	0.295	−2.714^**^
	(0.1242)	(0.0630)	(0.1960)	(0.0728)	(1.3816)	(0.0404)	(0.2934)	(0.3458)	(0.3908)	(0.5034)	(0.3677)	(5.5522)	(0.3463)	(1.1785)
Observations	13,567	15,155	10,685	10,999	10,608	9,011	6,542	10,567	12,006	7,716	8,613	8,496	7,607	4,950
*R*^2^/Pseudo *R*^2^	0.0784	0.0772	0.1055	0.0996	0.2037	0.1104	0.0614	0.1005	0.0954	0.1123	0.0978	0.2043	0.1285	0.0949

*** Significant at 1%;

** significant at 5%;

* significant at 10%.

*Clustered standard errors in parentheses, errors are clustered at the regions level*.

### Potential Channels

While the results uphold previous studies on the innovation–survival link, we further explore the potential mechanism through which innovative firms become more resilient to the crisis than non-innovators. We postulate that innovative firms are more adaptable to changes in the external environment than non-innovators, thus increase their survival chances. The study uses five measures as firm adaptation in response to the crisis: pivoting (adjusting production and services), introducing products/services in response to COVID-19 pandemic (Covidps), increased online activity, delivery of goods and services, and remote work arrangements. Innovation remains the key independent variable. The regression analysis uses the same set of firm-level control variables, the stringency of lockdown, country-industry fixed effects, and regions as a base for standard error clustering.

Results, reported in columns 1 to 5 of Table 4, confirm our conjecture that innovative firms are more likely to adapt to the external environment, affecting firm closures, expectations, and performance during bad times. More specifically, the results show that pre-crisis product innovation is positively related to pivoting, introducing new products/services, increased online activities, delivery, and remote work arrangements. These results are statistically significant across all estimated models. Thus, we suggest and find empirical support for the argument echo by previous studies that during recessions, innovators have better survival prospects than non-innovators owing to their ability to adapt and adjust to the changes in the external environment.

**Table 4:** Product innovation and adaptability.

	(1)	(2)	(3)	(4)	(5)
	Pivoting	Covidps	Online	Delivery	Remote
Product	0.180^***^	0.360^***^	0.250^***^	0.162^***^	0.159^***^
	(0.0420)	(0.0727)	(0.0417)	(0.0372)	(0.0394)
Medium	0.044	0.147^*^	0.103^**^	0.084^*^	0.278^***^
	(0.0466)	(0.0863)	(0.0496)	(0.0455)	(0.0460)
Large	0.093^*^	0.169^*^	0.081	0.056	0.574^***^
	(0.0483)	(0.1011)	(0.0570)	(0.0553)	(0.0590)
Training	0.178^***^	0.140^*^	0.018	0.039	0.166^***^
	(0.0354)	(0.0847)	(0.0355)	(0.0379)	(0.0364)
Productivity	−0.055^***^	0.005	0.017	−0.024	0.075^***^
	(0.0148)	(0.0249)	(0.0132)	(0.0156)	(0.0180)
Access Fin.	0.033	−0.000	−0.014	0.001	0.005
	(0.0208)	(0.0221)	(0.0163)	(0.0174)	(0.0140)
Quality	−0.099^**^	0.030	−0.051	−0.043	0.106^**^
	(0.0410)	(0.0915)	(0.0456)	(0.0555)	(0.0414)
Age	−0.082^***^	−0.125^***^	−0.042	−0.054^***^	0.013
	(0.0270)	(0.0401)	(0.0257)	(0.0208)	(0.0280)
Experience	0.006	0.025	−0.054^*^	−0.033	−0.084^***^
	(0.0252)	(0.0458)	(0.0308)	(0.0306)	(0.0277)
Website	0.057	0.059	0.338^***^	0.180^***^	0.149^***^
	(0.0363)	(0.0774)	(0.0412)	(0.0347)	(0.0414)
Foreign	−0.051	−0.001	0.013	−0.021	0.279^***^
	(0.0454)	(0.0914)	(0.0539)	(0.0780)	(0.0484)
Exporter	0.040	−0.016	−0.063	−0.181^***^	0.126^***^
	(0.0388)	(0.0644)	(0.0440)	(0.0493)	(0.0453)
Female Mang.	0.050	0.094^*^	0.002	0.084^**^	−0.027
	(0.0368)	(0.0563)	(0.0369)	(0.0394)	(0.0412)
Capital City	0.139^*^	0.199^*^	0.289^***^	0.117	0.188^**^
	(0.0737)	(0.1191)	(0.1059)	(0.0935)	(0.0899)
Stringency Index	0.008^**^	−0.000	−0.005	0.011^***^	−0.005
	(0.0034)	(0.0087)	(0.0045)	(0.0028)	(0.0039)
Industry F.E.	Yes	Yes	Yes	Yes	Yes
Region F.E.	Yes	Yes	Yes	Yes	Yes
_cons	0.085	−1.251^**^	−1.018^***^	−1.452^***^	−1.400^***^
	(0.3289)	(0.5534)	(0.3627)	(0.2855)	(0.2697)
Observations	9,029	3,104	8,685	9,045	9,020
*R*^2^/Pseudo *R*^2^	0.1217	0.0894	0.1239	0.1245	0.1372

*** Significant at 1%;

** significant at 5%;

* significant at 10%.

*Clustered standard errors in parentheses, errors are clustered at the regions level*.

To assess the robustness of our results suggesting firm adaptability as a potential mechanism for the innovation–survival relationship, we use an alternate definition of firm innovation. Table 5 presents the regression analysis results where we regressed pre-crisis process innovation against adaption variables and the same set of control variables. The results confirm our previous findings that being able to innovate constitutes a capability that leads firms to a greater fitness and better adapts to the environment, which ultimately increases firm survival.

**Table 5:** Process innovation and adaptability.

	(1)	(2)	(3)	(4)	(5)
	Pivoting	Covidps	Online	Delivery	Remote
Process	0.115^**^	0.156	0.110^**^	0.150^***^	0.140^***^
	(0.0497)	(0.1028)	(0.0548)	(0.0493)	(0.0532)
Medium	0.046	0.144^*^	0.100^**^	0.076^*^	0.272^***^
	(0.0463)	(0.0872)	(0.0502)	(0.0459)	(0.0467)
Large	0.101^**^	0.165	0.079	0.051	0.572^***^
	(0.0486)	(0.1030)	(0.0579)	(0.0551)	(0.0591)
Training	0.185^***^	0.171^**^	0.036	0.044	0.170^***^
	(0.0357)	(0.0870)	(0.0363)	(0.0391)	(0.0372)
Productivity	−0.056^***^	0.005	0.016	−0.024	0.076^***^
	(0.0146)	(0.0250)	(0.0136)	(0.0160)	(0.0179)
Access Fin.	0.032	−0.002	−0.016	0.002	0.004
	(0.0210)	(0.0229)	(0.0164)	(0.0175)	(0.0141)
Quality	−0.101^**^	0.035	−0.054	−0.048	0.100^**^
	(0.0412)	(0.0898)	(0.0457)	(0.0557)	(0.0415)
Age	−0.084^***^	−0.126^***^	−0.042^*^	−0.051^**^	0.013
	(0.0266)	(0.0412)	(0.0249)	(0.0208)	(0.0280)
Experience	0.003	0.015	−0.058^*^	−0.039	−0.083^***^
	(0.0254)	(0.0434)	(0.0305)	(0.0305)	(0.0279)
Website	0.068^*^	0.090	0.359^***^	0.192^***^	0.155^***^
	(0.0358)	(0.0772)	(0.0411)	(0.0348)	(0.0411)
Foreign	−0.060	0.000	0.001	−0.028	0.277^***^
	(0.0459)	(0.0904)	(0.0547)	(0.0778)	(0.0477)
Exporter	0.049	0.015	−0.050	−0.173^***^	0.140^***^
	(0.0385)	(0.0637)	(0.0424)	(0.0489)	(0.0446)
Female Mang.	0.055	0.105^*^	0.012	0.091^**^	−0.020
	(0.0374)	(0.0564)	(0.0369)	(0.0399)	(0.0410)
Capital City	0.144^*^	0.224^*^	0.296^***^	0.126	0.198^**^
	(0.0741)	(0.1174)	(0.1079)	(0.0939)	(0.0914)
Stringency Index	0.008^**^	−0.001	−0.006	0.011^***^	−0.005
	(0.0036)	(0.0087)	(0.0043)	(0.0027)	(0.0039)
Industry F.E.	Yes	Yes	Yes	Yes	Yes
Region F.E.	Yes	Yes	Yes	Yes	Yes
_cons	0.139	−1.142^**^	−0.912^***^	−1.439^***^	−1.383^***^
	(0.3485)	(0.5731)	(0.3499)	(0.2903)	(0.2698)
Observations	9,022	3,099	8,677	9,037	9,012
*R*^2^/Pseudo *R*^2^	0.1202	0.0803	0.1194	0.1194	0.1366

*** Significant at 1%;

** significant at 5%;

* significant at 10%.

*Clustered standard errors in parentheses, errors are clustered at the regions level*.

## Discussion and Conclusion

In general, firms play an important role in economic development. Aside from the government, many other stakeholders in the community stand to gain from firm survival. They include managers, employees, consumers, and suppliers (Bercovitz and Mitchell, 2007). However, firms’ survival is endangered during times of crisis (Bosio et al., 2020). Crises have a detrimental impact on the growth of businesses and their projects because the negative impact of crisis extends to all elements of the external business environment (Dhochak and Sharma, 2015). Firms have limited funding options and a greater probability of failures due to poor capital market performance during a crisis, lack of sufficient information, and component defects throughout the economy (Chundakkadan et al., 2020; Gormsen and Koijen, 2020;McKibbin and Fernando, 2021).

During this recent COVID-19 crisis, firms faced difficulties in performing their operational activities, severe liquidity constraints, and, ultimately, risk of failure (Apedo-Amah et al., 2020; Bartik et al., 2020; Dai et al., 2020b). The rapidly growing literature on COVID-19 sheds initial light on firm-specific characteristics that have enabled firms to cope with and survive the shock (Ding et al., 2020; Guo et al., 2020; Li et al., 2020; Grover and Karplus, 2021) but thus far none has focused on innovation practices. Therefore, consistent with this stream of literature, the main purpose of our study is to highlight the importance of innovation practices as a response to the COVID-19 crisis and its effect on business performance and the likelihood of their survival.

For any business, innovation is critical to its long-term success (Zhang et al., 2018). Innovation, according to Gaynor (2002), is a critical factor in a firm’s long-term survival and performance. It helps an enterprise to expand and grow while also enhancing its future success. Firms can succeed and survive by using innovations to overcome obstacles and challenges. Prior studies have shown that firm innovation practices are a significant determinant of firm performance and survival during crisis and non-crisis situation (Yıldız et al., 2014; Fernandes and Paunov, 2015; Oura et al., 2016; Cefis et al., 2020; Fiorentino et al., 2020; Ugur and Vivarelli, 2021).

Using a sample of 15,451 firms in 27 countries, we find that innovation is negatively related to permanent firm’s closures during the COVID-19 crisis. This is also supported by evidence that innovative firms performed better than non-innovators during the crisis and were also optimistic about future economic conditions. Our study’s most striking finding is that the innovation relationship to survival outcomes runs strongly and primarily through adaptability. We provide strong evidence that innovative firms are more likely to adapt to new changes in the external environment, thus increasing their survival prospects. Our research shows that innovators not only fare better through crises just because they are innovators but also because they can better adapt to new developments, which increases the likelihood of survival for innovators.

The study’s findings have significant managerial and policy implications. To begin, crises typically affect an organization’s sales, production capabilities, and financial conditions; therefore, managers should continue to develop and support innovation in relation to all business activities to meet the challenges imposed by the pandemic. Second, managers should stay up-to-date on knowledge and information solutions that will assist them in making sound decisions and getting through the crisis successfully. It is also crucial that managers update their plans and strategies regularly to achieve the flexibility required to respond to the new conditions in the external environment. Further, the post-pandemic state of businesses will be very different from that of pre-pandemic businesses, therefore, managers should develop a strategy to deal with the crisis’ negative effects on their businesses after the pandemic to ensure continuity and survival.

Although the current study has achieved findings that have significant implications for managers and policymakers, it has some limitations. The paper uses pre and post COVID-19 firm-level data from the World Bank Enterprise Surveys. The advantage of using a database such as WBES is that it enables relatively large sample sizes to be used for statistical analysis. However, the main disadvantage is that one has no control over the specific variables that are available for inclusion in the study. Since this research is based on a large set of cross-sectional data, future research can find out if the results are similar to longitudinal.

## Data Availability Statement

Publicly available datasets were analyzed in this study. This data can be found here: The datasets used or analyzed during the current study are available on the data portal of the World Bank Enterprise Surveys. https://login.enterprisesurveys.org/content/sites/financeandprivatesector/en/signin.html?deliveryName=DM102619.

## Author Contributions

All authors listed have made a substantial, direct, and intellectual contribution to the work and approved it for publication.

## Author Disclaimer

The results and conclusions discussed in this paper are solely the responsibility of the authors, and do not necessarily reflect the views of the World Bank. Responsibility for any errors are with the authors.

## Conflict of Interest

The authors declare that the research was conducted in the absence of any commercial or financial relationships that could be construed as a potential conflict of interest.

## Publisher’s Note

All claims expressed in this article are solely those of the authors and do not necessarily represent those of their affiliated organizations, or those of the publisher, the editors and the reviewers. Any product that may be evaluated in this article, or claim that may be made by its manufacturer, is not guaranteed or endorsed by the publisher.

## Footnotes

^1^Source: WHO Coronavirus Dashboard Website https://covid19.who.int/?gclid=CjwKCAiAqJn9BRB0EiwAJ1SztfTqbKTY7auGN8lsfHZpFQj5NLSMFGIl_mrPddCUv50KOwW_RLmOFhoCEBsQAvD_BwE

^2^Source: Our World in Data https://ourworldindata.org/grapher/covid-stringency-index

^3^Regions within country.

^4^Hudson et al. (2012) and Nichols and Schaffer (2007) notes that small number of clusters, such as fewer than 50, or very unbalanced cluster sizes may cause more problems than it solves. Both conditions, in our case, mitigates the use of countries as base for cluster and satisfies the use of regions as base for clustering errors.

## References

[ref1] Adam N. A. Alarifi G. (2021). Innovation practices for survival of small and medium enterprises (SMEs) in the COVID-19 times: the role of external support. J. Innovation Entrepreneurship 10:15. doi: 10.1186/s13731-021-00156-6 PMC8155652 34075328

[ref2] Apedo-Amah M. C. Avdiu B. Cirera X. Cruz M. Davies E. Grover A.. (2020). Unmasking the impact of COVID-19 on businesses: firm level evidence from across the world, unmasking the impact of COVID-19 on businesses: firm level evidence from across the world. Available at: 10.1596/1813-9450-9434 (Accessed June 2021).

[ref3] Balasubramanian N. Lee J. (2008). Firm age and innovation. Ind. Corp. Chang. 17, 1019–1047. doi: 10.1093/icc/dtn028

[ref4] Barney J. (1991). Firm resources and sustained competitive advantage. J. Manag. 17, 99–120. doi: 10.1177/014920639101700108

[ref5] Bartik A. W. Bertrand M. Cullen Z. Glaeser E. L. Luca M. Stanton C. (2020). The impact of COVID-19 on small business outcomes and expectations. Proc. Natl. Acad. Sci. 117, 17656–17666. doi: 10.1073/pnas.2006991117 32651281 PMC7395529

[ref001] Baumol W. J. (2002). The Free-Market Innovation Machine: Analyzing the Growth Miracle of Capitalism. Princeton: Princeton University Press.

[ref6] Bello-Pintado A. Bianchi C. (2018). Educational diversity, organizational structure and innovation performance: evidence from Uruguayan industry. Estudios de Economía 35, 203–229.

[ref7] Bercovitz J. Mitchell W. (2007). When is more better? The impact of business scale and scope on long-term business survival, while controlling for profitability. Strateg. Manag. J. 28, 61–79. doi: 10.1002/smj.568

[ref8] Børing P. Fevolden A. M. Herstad S. (2016). Eager and able: a study of innovation activity among young, mature and old firms in Norway. Econ. Bull. 36, 291–287.

[ref9] Bosio E. Djankov S. Jolevski F. Ramalho R. (2020). Survival of firms during economic crisis. SSRN Electron. J. doi: 10.2139/ssrn.3588346

[ref10] Bratti M. Felice G. (2012). Are exporters more likely to introduce product innovations? World Econ. 35, 1559–1598. doi: 10.1111/j.1467-9701.2012.01453.x

[ref11] Cefis E. (2003). Is there persistence in innovative activities? Int. J. Ind. Organ. 21, 489–515. doi: 10.1016/S0167-7187(02)00090-5

[ref12] Cefis E. (2005). A matter of life and death: innovation and firm survival. Ind. Corp. Chang. 14, 1167–1192. doi: 10.1093/icc/dth081

[ref13] Cefis E. Bartoloni E. Bonati M. (2020). Show me how to live: firms’ financial conditions and innovation during the crisis. Struct. Chang. Econ. Dyn. 52, 63–81. doi: 10.1016/j.strueco.2019.10.001

[ref14] Cefis E. Marsili O. (2006). Survivor: the role of innovation in firms’ survival. Res. Policy 35, 626–641. doi: 10.1016/j.respol.2006.02.006

[ref15] Cefis E. Marsili O. (2011). Born to flip. Exit decisions of entrepreneurial firms in high-tech and low-tech industries. J. EEcon. 21, 473–498. doi: 10.1007/s00191-010-0210-4

[ref16] Cefis E. Marsili O. (2012). Going, going, gone. Exit forms and the innovative capabilities of firms. Res. Policy 41, 795–807. doi: 10.1016/j.respol.2012.01.006

[ref17] Cefis E. Marsili O. (2019). Good times, bad times: innovation and survival over the business cycle. Ind. Corp. Chang. 28, 565–587. doi: 10.1093/icc/dty072

[ref18] Chandran Govindaraju V. G. R. Krishnan Vijayaraghavan G. Pandiyan V. (2013). Product and process innovation in Malaysian manufacturing: the role of government, organizational innovation and exports. Innovations 15, 52–68. doi: 10.5172/impp.2013.15.1.52

[ref19] Chundakkadan R. Raj R. Sasidharan S. (2020). Small firms amidst COVID-19: financial constraints and role of government support. SSRN Electron. J. doi: 10.2139/ssrn.3691564

[ref20] Coad A. Guenther C. (2013). Diversification patterns and survival as firms mature. Small Bus. Econ. 41, 633–649. doi: 10.1007/s11187-012-9447-7

[ref21] Cohen W. M. Klepper S. (1996a). Firm size and the nature of innovation within industries: the case of process and product R&D. Rev. Econ. Stat. 78:232. doi: 10.2307/2109925

[ref22] Cohen W. M. Klepper S. (1996b). A reprise of size and R & D. Econ. J. 106:925. doi: 10.2307/2235365

[ref23] Colombelli A. Krafft J. Quatraro F. (2013). Properties of knowledge base and firm survival: evidence from a sample of French manufacturing firms. Technol. Forecast. Soc. Chang. 80, 1469–1483. doi: 10.1016/j.techfore.2013.03.003

[ref24] Dai R. Feng H. Hu J. Jin Q. Li H. Wang R.. (2020b). The impact of COVID-19 on small and medium-sized enterprises: evidence from two-wave phone surveys in China. Working Paper 67:101607. doi: 10.1016/j.chieco.2021.101607 PMC9761881 36568286

[ref25] Dai M. Liu H. Lin L. (2020a). How innovation impacts firms’ export survival: does export mode matter? World Econ. 43, 81–113. doi: 10.1111/twec.12847

[ref26] Dhochak M. Sharma A. K. (2015). Impact of global financial crisis on Indian venture capital firms: an empirical evaluation. J. Int. Bus. Entrepreneurship Dev. 8:330. doi: 10.1504/JIBED.2015.072931

[ref27] Ding W. Levine R. Lin C. Xie W. (2020). Corporate immunity to the COVID-19 pandemic. Nat. Bur. Econ. Res. doi: 10.3386/w27055 PMC8457922 34580557

[ref28] Esteve-Pérez S. Mañez-Castillejo J. A. (2008). The resource-based theory of the firm and firm survival. Small Bus. Econ. 30, 231–249. doi: 10.1007/s11187-006-9011-4

[ref29] Fernandes A. M. Paunov C. (2015). The risks of innovation: are innovating firms less likely to die? Rev. Econ. Stat. 97, 638–653. doi: 10.1162/REST_a_00446

[ref30] Fiorentino R. Longobardi S. Scaletti A. (2020). The early growth of start-ups: innovation matters. Evidence from Italy. Eur. J. Innov. Manag. 24, 1525–1546. doi: 10.1108/EJIM-02-2020-0057

[ref31] Fontana R. Nesta L. (2009). Product innovation and survival in a high-tech industry. Rev. Ind. Organ. 34, 287–306. doi: 10.1007/s11151-009-9210-7

[ref32] Fu M. Shen H. (2020). COVID-19 and corporate performance in the energy industry. Energy Res. Lett. doi: 10.46557/001c.12967

[ref33] Garriga H. von Krogh G. Spaeth S. (2013). How constraints and knowledge impact open innovation. Strateg. Manag. J. 34, 1134–1144. doi: 10.1002/smj.2049

[ref34] Gaynor G. H. (2002). Innovation by Design: What it Takes to Keep your Company on the Cutting Edge. New York: AMACOM.

[ref35] Gormsen N. J. Koijen R. S. J. (2020). “Coronavirus: impact on stock prices and growth expectations,” edited by Roussanov, N. Rev Asset Pric Stud 10, 574–597. doi: 10.1093/rapstu/raaa013

[ref36] Grover A. Karplus V. J. (2021). Coping with COVID-19 does management make firms more resilient? No. 9514; Policy Research Working Paper. Available at: https://hdl.handle.net/10986/35028 (Accessed April 2021).

[ref37] Guo H. Yang Z. Huang R. Guo A. (2020). The digitalization and public crisis responses of small and medium enterprises: implications from a COVID-19 survey. Front. Bus. Res. China. doi: 10.1186/s11782-020-00087-1

[ref38] Gupta A. (2020). R&D and firm resilience during bad times. SSRN Electron. J. doi: 10.2139/ssrn.3703103

[ref39] Hansen J. A. (1992). Innovation, firm size, and firm age. Small Bus. Econ. 4, 37–44.

[ref40] Helmers C. Rogers M. (2010). Innovation and the survival of new firms in the UK. Rev. Ind. Organ. 36, 227–248. doi: 10.1007/s11151-010-9247-7

[ref41] Hudson J. Williams C. Orviska M. Nadin S. (2012). Evaluating the impact of the informal economy on businesses in south East Europe: some lessons from the 2009 World Bank Enterprise survey. South East Eur. J. Econ. Bus. 7, 99–110. doi: 10.2478/v10033-012-0010-x

[ref42] Khan K. Li Y. Liu S. Li C. (2021). Psychological distress and trust in university management among international students during the COVID-19 pandemic. Front. Psychol. 12:679661. doi: 10.3389/fpsyg.2021.679661 34220649 PMC8250427

[ref43] Khan K. Zhao H. Zhang H. Yang H. Shah M. H. Jahanger A. (2020). “The impact of COVID-19 pandemic on stock markets: an empirical analysis of world major stock indices.” J. Asian Finan. Econ. Bus, 7, 463–474, doi: 10.13106/jafeb.2020.vol7.no7.463

[ref44] Kotha R. Zheng Y. George G. (2011). Entry into new niches: the effects of firm age and the expansion of technological capabilities on innovative output and impact. Strategic Manage. J. 32, 1011–1024. doi: 10.1002/smj.915

[ref45] Li K. Liu X. Mai F. Zhang T. (2020). The role of corporate culture in bad times: evidence from the COVID-19 pandemic. SSRN Electron. J. doi: 10.2139/ssrn.3632395

[ref46] Love J. H. Roper S. Vahter P. (2014). Learning from openness: the dynamics of breadth in external innovation linkages. Strateg. Manag. J. 35, 1703–1716. doi: 10.1002/smj.2170

[ref002] Lundvall B. Å. (1988). “Innovation as an interactive process: from user-producer interaction to the national system of innovation,” in Technical Change and Economic Theory. eds. Dosi G. Freeman C. Nelson R. Silverberg G. Soete L. (Pinter, London), 349–369.

[ref47] McKibbin W. Fernando R. (2021). The global macroeconomic impacts of COVID-19: seven scenarios. Asian Econ. Papers 20, 1–30. doi: 10.1162/asep_a_00796

[ref48] Monreal-Pérez J. Aragón-Sánchez A. Sánchez-Marín G. (2012). A longitudinal study of the relationship between export activity and innovation in the Spanish firm: the moderating role of productivity. Int. Bus. Rev. 21, 862–877. doi: 10.1016/j.ibusrev.2011.09.010

[ref49] Narayan P. K. (2020). Oil price news and COVID-19—is there any connection? Energy Res. Lett. doi: 10.46557/001c.13176

[ref50] Nelson R. R. Winter S. G. (1982). An Evolutionary Theory of Economic Change. Harvard University Press: Cambridge, MA.

[ref51] Nichols A. Schaffer M. (2007). “Clustered errors in Stata,” United Kingdom Stata Users’ Group Meetings. Available at: http://www.stata.com/meeting/13uk/nichols_crse.pdf (Accessed June 2021).

[ref52] Oura M. M. Zilber S. N. Lopes E. L. (2016). Innovation capacity, international experience and export performance of SMEs in Brazil. Int. Bus. 25, 921–932. doi: 10.1016/j.ibusrev.2015.12.002

[ref53] Porter M.E. (1980). Competitive Strategy: Techniques for Analyzing Industries and Competitors. New York, USA:The Free Press.

[ref54] Reichstein T. Salter A. (2006). Investigating the sources of process innovation among UK manufacturing firms. Ind. Corporate Change 15, 653–682. doi: 10.1093/icc/dtl014

[ref55] Reinhart C. M. (2020). “This Time Truly Is Different.” Available at: https://www.project-syndicate.org/commentary/covid19-crisis-has-no-economic-precedent-by-carmen-reinhart-2020-03?barrier=accesspaylog (Accessed June 2021).

[ref56] Ren S. Eisingerich A. B. Tsai H.-T. (2015). How do marketing, research and development capabilities, and degree of internationalization synergistically affect the innovation performance of small and medium-sized enterprises (SMEs)? A panel data study of Chinese SMEs. Int. Bus. Rev. 24, 642–651. doi: 10.1016/j.ibusrev.2014.11.006

[ref57] Rosenbusch N. Brinckmann J. Bausch A. (2011). Is innovation always beneficial? A meta-analysis of the relationship between innovation and performance in SMEs. J. Bus. Venturing 26, 441–457. doi: 10.1016/j.jbusvent.2009.12.002

[ref58] Schumpeter J. A. (1943). Capitalism, Socialism and Democracy. New York: Harper.

[ref59] Seenaiah K. Rath B. N. (2018). Determinants of innovation in selected manufacturing firms in india: role of R&D and exports. Sci. Technol. Soc. 23, 65–84. doi: 10.1177/0971721817744445

[ref60] Sidorkin O. Srholec M. (2014). Surviving the times of crisis: does innovation make a difference? Int. J. Technol. Learn. Innovation Dev. 7, 124–84. doi: 10.1504/IJTLID.2014.065881

[ref61] Song M. Podoynitsyna K. Van Der Bij H. Halman J. I. M. (2007). Success factors in new ventures: a meta-analysis*. J. Prod. Innov. Manag. 25, 7–27. doi: 10.1111/j.1540-5885.2007.00280.x

[ref62] Spithoven A. Vanhaverbeke W. Roijakkers N. (2013). Open innovation practices in SMEs and large enterprises. Small Bus. Econ. 41, 537–562. doi: 10.1007/s11187-012-9453-9

[ref63] Sun Y. Du D. (2010). Determinants of industrial innovation in China: evidence from its recent economic census. Technovation 30, 540–550. doi: 10.1016/j.technovation.2010.05.003

[ref64] Teece D. J. Pisano G. Shuen A. (1997). Dynamic capabilities and strategic management. Strateg. Manag. J. 18, 509–533. doi: 10.1002/(SICI)1097-0266(199708)18:7<509::AID-SMJ882>3.0.CO;2-Z

[ref65] Ugur M. Vivarelli M. (2021). Innovation, firm survival and productivity: the state of the art. Econ. Innov. New Technol. 30, 433–467. doi: 10.1080/10438599.2020.1828509

[ref66] Wagner S. Cockburn I. (2010). Patents and the survival of internet-related IPOs. Res. Policy 39, 214–228. doi: 10.1016/j.respol.2009.12.003

[ref003] Withers M. C. Drnevich P. L. Marino L. (2011). Doing more with less: the disordinal implications of firm age for leveraging capabilities for innovation activity. J. Small Bus. Manag. 49, 515–536. doi: 10.1111/j.1540-627X.2011.00334.x

[ref67] Yıldız S. Baştürk F. Boz İ. T. (2014). The effect of leadership and innovativeness on business performance. Procedia. Soc. Behav. Sci. 150, 785–793. doi: 10.1016/j.sbspro.2014.09.064

[ref68] Zahra S. A. George G. (2002). Absorptive capacity: a review, reconceptualization, and extension. Acad. Manag. Rev. 27, 185–203. doi: 10.2307/4134351

[ref69] Zhang D. Zheng W. Ning L. (2018). Does innovation facilitate firm survival? Evidence from Chinese high-tech firms. Econ. Model. 75, 458–468. doi: 10.1016/j.econmod.2018.07.030

